# Biomimetic Transfer Learning-Based Complex Gastrointestinal Polyp Classification

**DOI:** 10.3390/biomimetics10100699

**Published:** 2025-10-15

**Authors:** Daniela-Maria Cristea, Daniela Onita, Laszlo Barna Iantovics

**Affiliations:** 1 Department of Computer Science and Engineering, ‘1 Decembrie 1918’ University of Alba Iulia, 510009 Alba Iulia, Romania; daniela.onita@uab.ro; 2 Doctoral School of Letters, Humanities and Applied Sciences, George Emil Palade University of Medicine, Pharmacy, Sciences and Technology of Targu Mures, 540142 Targu Mures, Romania; 3 Electrical Engineering and Information Technology Department, George Emil Palade University of Medicine, Pharmacy, Sciences and Technology of Targu Mures, 540142 Targu Mures, Romania

**Keywords:** artificial neural network, biomimetic algorithm, medical imaging, computational hard problem, gastrointestinal polyps, machine learning, deep neural network, colorectal disease

## Abstract

(1) Background: This research investigates the application of Artificial Intelligence (AI), particularly biomimetic convolutional neural networks (CNNs), for the automatic classification of gastrointestinal (GI) polyps in endoscopic images. The study combines AI and Transfer learning techniques to support early detection of colorectal cancer by enhancing diagnostic accuracy with pre-trained models; (2) Methods: The Kvasir dataset, comprising 4000 annotated endoscopic images across eight polyp categories, was used. Images were pre-processed via normalisation, resizing, and data augmentation. Several CNN architectures, including state-of-the-art optimized ResNet50, DenseNet121, and MobileNetV2, were trained and evaluated. Models were assessed through training, validation, and testing phases, using performance metrics such as overall accuracy, confusion matrix, precision, recall, and F1 score; (3) Results: ResNet50 achieved the highest validation accuracy at 90.5%, followed closely by DenseNet121 with 87.5% and MobileNetV2 with 86.5%. The models demonstrated good generalisation, with small differences between training and validation accuracy. The average inference time was under 0.5 s on a computer with limited resources, confirming real-time applicability. Confusion matrix analysis indicates that common errors frequently occur between visually similar classes, particularly when reviewed by less-experienced medical physicians. These errors underscore the difficulty of distinguishing subtle features in gastrointestinal imagery and highlight the value of model-assisted diagnostics; (4) Conclusions: The obtained results confirm that Deep learning-based CNN architectures, combined with Transfer learning and optimisation techniques, can classify accurately endoscopic images and support medical diagnostics.

## 1. Introduction

Colorectal cancer (also known as colon or rectal cancer) is one of the most common causes of death worldwide, and detecting gastrointestinal (GI) polyps plays an important role in its prevention [1,2]. With the rapid advancement of Artificial Intelligence (AI) and neural networks, the application of these technologies in the medical field is becoming increasingly promising. Certain GI polyps, particularly adenomatous and serrated types, carry a risk of progressing to cancer if not identified and removed in time. Regular screening and timely intervention are essential to reduce the likelihood of malignant transformation. Therefore, integrating AI into diagnostic processes can provide significant support for physicians, enabling them to identify errors more quickly and accurately. The goal is not to replace human expertise but to complement it by providing specialists with modern tools that enhance their work and reduce human error rates [3].

Compared to classical Machine Learning (ML) methods [4,5], which perform well with structured and diverse datasets, Deep Learning (DL) architectures [6,7], like artificial neural networks, are themselves biomimetic in nature, modelled after the human brains layered processing, making them powerful for interpreting biological data in domains such as medical image diagnostics or facial recognition [8].

Artificial Neural Networks (ANNs) [9] are an important subset of ML algorithms, inspired by the structure and functioning of the human brain [10]. They consist of layers of artificial neurons that process data through weighted connections, allowing the system to learn and generalize from complex data. Convolutional Neural Networks (CNNs) [11] are a specialized category of artificial neural networks, primarily used in image processing and automatic visual pattern recognition. These networks are inspired by how the human brain processes visual information, enabling them to learn spatial and hierarchical features from raw images [12,13].

A CNN structure includes several types of layers: convolutional layers, activation layers (e.g., ReLU), pooling layers (e.g., max-pooling), followed by one or more fully connected layers. Convolutional layers apply filters (kernels) over images, automatically extracting important features such as edges, complex shapes, or text regions [14].

CNNs have rapidly evolved due to their ability to automatically extract visual features without human intervention. Initially, models like Alex Convolutional Neural Network (AlexNet) [13], Visual Geometry Group Network (VGGNet) [14], and Inception Network by Google (GoogleNet) [15] were used for simple binary classification tasks.

Later, deeper networks such as Residual Network (ResNet) [16] and Densely Connected Convolutional Network (DenseNet) [17] improved the classification, detection, and segmentation of brain lesions, including gliomas, meningiomas, and tumors, in multimodal Magnetic Resonance Imaging (MRI) scans such as T1-weighted, T2-weighted, Fluid Attenuated Inversion Recovery (FLAIR), and contrast-improved sequences [18].

Recent research has focused on hybrid neural architectures such as Convolutional Neural Network-Long Short-Term Memory (CNN-LSTM) models, which combine spatial feature extraction with temporal pattern recognition. These models are particularly effective in medical imaging tasks where both spatial and sequential data characteristics are present. Transfer learning techniques have also gained traction, leveraging pre-trained models like VGGNet, ResNet, and Inception trained on large-scale datasets such as ImageNet (over 14 million images) [19,20] and adapting them to smaller medical datasets, often containing fewer than 10,000 annotated images. This approach obtained improvements in classification accuracy, sensitivity, and specificity, especially in tasks like brain tumor grading, breast cancer subtype identification, and lesion segmentation in MRI and histopathology images [21].

In medical imaging, transparency in AI decisions is crucial. Explainable Artificial Intelligence (XAI) techniques such as Gradient-weighted Class Activation Mapping (Grad-CAM), Local Interpretable Model-Agnostic Explanations (LIME), and SHapley Additive exPlanations (SHAP) enable visualization of image regions that significantly influence model predictions, helping radiologists interpret how decisions are made [22]. The literature study [9] contributes to the evolving field of AI-driven medical image analysis by comparing multiple CNN architectures [6], both classical and advanced, including Capsule Network (CapsNet) and FastAI-based models. These architectures are applied to a specialized endoscopic image dataset. Studies highlight the applicability of Grad-CAM in localizing suspicious lesions in mammograms or Computed Tomography (CT) scans, while LIME and SHAP have been successfully used in predicting COVID-19 severity from lung radiographs [18].

Numerous studies have demonstrated the efficiency of CNNs in medical image analysis, especially in detecting and classifying anomalies in endoscopic procedures. For instance, the study [23] introduced the Kvasir dataset and evaluated several automatic classification methods for GI images, providing an essential starting point for performing research.

This research presents an integrative deep learning framework of advanced CNN architectures for the automatic classification of gastrointestinal lesions from endoscopic images. The proposed methodology integrates biomimetic modeling with Explainable Artificial Intelligence (XAI) techniques, including Grad-CAM, LIME, and SHAP.

The architectures evaluated in this work include classical models such as AlexNet and VGGNet, as well as more advanced frameworks like Inception Network (GoogLeNet), Residual Network (ResNet), Densely Connected Convolutional Network (DenseNet), and Capsule Network (CapsNet). These models were benchmarked using the Kvasir dataset [23] and Transfer learning [5] is employed to adapt large-scale pre-trained models. The classes used represent a mix of anatomical landmarks, pathological findings, and procedural outcomes and are defined as follows:

- Dyed-lifted polyps: Images showing polyps that have been stained and elevated using submucosal injection, aiding in visual contrast and resection planning;
- Dyed-resection-margins: Post-polypectomy images highlighting the margins of resected areas, stained to assess completeness of removal;
- Esophagitis: Inflammatory lesions of the esophageal mucosa, often appearing as erythematous streaks or erosions near the Z-line;
- Polyps: Unstained mucosal protrusions, typically benign growths that may serve as precursors to colorectal cancer;
- Ulcerative-colitis: Chronic inflammatory changes in the colon, characterized by mucosal ulceration, granularity, and vascular pattern loss;
- Normal-cecum: Anatomical landmark at the beginning of the large intestine, often used to confirm a complete colonoscopy;
- Normal-pylorus: The muscular opening between the stomach and duodenum, appearing as a round, symmetric structure in healthy individuals;
- Normal-z-line: The gastroesophageal junction, where the squamous epithelium of the esophagus transitions to the columnar epithelium of the stomach.

The novelty of this study lies in the fact that it explores a biomimetic approach to model design, inspired by biological principles such as layered abstraction and feature reuse, used to evaluate and compare advanced CNN architectures. These principles are reflected in the selection of architectures like ResNet50, DenseNet121, and CapsuleNet, which simulate cognitive processes through residual connections, dense feature propagation, and attention mechanisms. The goal is to identify the computationally most efficient model for the automatic classification of gastrointestinal lesions in endoscopic images, as applied to the Kvasir dataset.

The main contributions of this study to the state-of-the-art in medical image classification are as follows:

- Benchmarking of CNN Architectures: A set of CNN models, including classical architectures like AlexNet and VGGNet, and advanced designs such as GoogLeNet (Inception), ResNet, DenseNet, and CapsNet, were tested on the Kvasir dataset [23], composed of real-world endoscopic images, to assess their effectiveness in gastrointestinal lesion categorization.
- Optimizations: DenseNet121 and ResNet50 were fine-tuned using Transfer learning and dynamic class weighting, while CapsNet was improved with attention mechanisms to improve feature localization and reduce overfitting, especially in classes with limited samples. Thus, choosing ResNet50 represents a contribution of this study, guided by both optimization parameter tuning and empirical performance metrics, including validation accuracy and loss behavior across multiple folds.
- Biomimetic Model Selection: The study introduces a biomimetic framework for selecting CNN architectures, inspired by the hierarchical and layered processing of the human visual cortex [12]. This approach guided the prioritisation of models that emulate biological feature abstraction, such as residual and capsule-based networks.
- Explainability Integration: To improve performance on sparse and imbalanced medical datasets, this study combines Explainable Artificial Intelligence (XAI) techniques, namely Grad-CAM, LIME, and SHAP, with Transfer learning from large-scale datasets such as ImageNet. The novelty lies in the adaptation of XAI methods to guide model and error analysis.
- Benchmarking: The comparative analysis between ResNet50 [16], DenseNet121 [17], and MobileNetV2 [24] offered the best trade-off between accuracy, inference speed, and generalisation. In contrast, deeper models such as NASNetLarge and EfficientNetB8 showed signs of overfitting and slower inference.

The upcoming structure of the work is divided into five chapters. Section 1 Introduction provides a general introduction to the topic, explains why the subject was chosen, and presents the current context of AI in the medical field. It also describes general objectives, methodology, and the dataset used. Section 2 Materials and Methods covers theoretical foundations of machine learning and neural networks, focusing on CNNs and Transfer learning, with details on the CNN architectures used, describes their selection process, and mentions models excluded after preliminary testing. It also explains the research methodology, dataset, experimental stages, infrastructure, and applied evaluation methods. Section 3 Results presents accuracy and loss graphs for each tested CNN architecture. These graphs illustrate the model’s behavior during training and validation. Section 4 Discussion and Section 5 Conclusions highlight observed limitations, include case studies of applications of CNNs in medical practice, and conclude the paper with a summary of findings and possible directions for future studies.

## 2. Materials and Methods

Initially, a viable baseline was prepared: ResNet50 pretrained on ImageNet, using standard augmentations (rotation, flipping, zoom) and Keras’s *preprocess_input()* function to ensure compatibility with pretrained weights. This configuration served as a reference point for later evaluating more complex architectures [25,26].

Model Selection. DenseNet121 was selected for its feature reuse mechanism, which promotes efficient learning and reduces overfitting. MobileNetV2 was included for its design, and CapsuleNet was added to test robustness against pose variation, particularly in classes with high intra-class variability.

The dataset contains 500 images per class across eight classes, forming a strictly balanced distribution. However, training revealed the presence of hard examples, images with poor lighting, ambiguous textures, or overlapping features. To address this, we implemented a dynamic reweighting strategy based on sample difficulty rather than static class frequency. This approach ensures that harder examples receive more attention during training, improving generalization. All models used architecture-specific preprocessing functions from Keras. The experiments were conducted on an NVIDIA GeForce RTX 3050 (4 GB GDDR6) GPU with the following training parameters: 32 batch size, Adam optimizer, 0.001 the initial learning rate, 0.0001 the minimum learning rate, 0.00001 weight decay, 10 epochs for early stopping patience.

A graphical user interface (GUI) was developed using the Python (v3.13) Tkinter library, integrated with TensorFlow Keras models. The GUI allows users to upload images, select models, and view predictions, facilitating clinical usability.

### 2.1. Dataset and Methodology

For this research, a public dataset called Kvasir [23] was used, a Multi-Class Image Dataset for Computer-Aided GI Disease Detection, containing 4000 GI images divided into eight polyp classes. It was created for research purposes to develop automatic disease detection algorithms for gastrointestinal conditions. The dataset was published by Simula Research Laboratory in collaboration with Vestre Viken Health Trust in Norway, a network of four hospitals that provides medical care to approximately 470,000 people [27].

The dataset includes manually annotated and verified images from real diagnoses by endoscopy specialists. The images originate from actual diagnostic procedures and include a variety of anatomical landmarks, pathological findings, or endoscopic procedures observed in upper digestive tract examinations.

This dataset consists of two files: one file containing eight classes of polyp types: Dyed-lifted-polyps, Dyed-resection-margins, Esophagitis, Polyps, Ulcerative-colitis, Normal-cecum, Normal-pylorus, Normal-z-line. Each class has 500 images covering key categories such as polyps, ulcers, esophagus conditions, hemorrhages, and others, with each class containing 500 images of variable dimensions and different lighting conditions. In addition to raw images, the dataset includes a separate file containing extracted features for each image [28,29].

### 2.2. Dataset Collection and Annotation

The images in the Kvasir dataset were collected under real clinical conditions at partnering gastroenterology centers. Each image is accompanied by expert-level annotations, performed in collaboration with certified endoscopy specialists and validated by the research team involved in the study presented in Ref. [29]. All images were uniformly scaled during our study to 224 × 224 pixels (or 299 × 299 for Inception-based models), enabling standardised input dimensions for CNN-based analysis and preserving spatial integrity across architectures.

**Figure 1:**
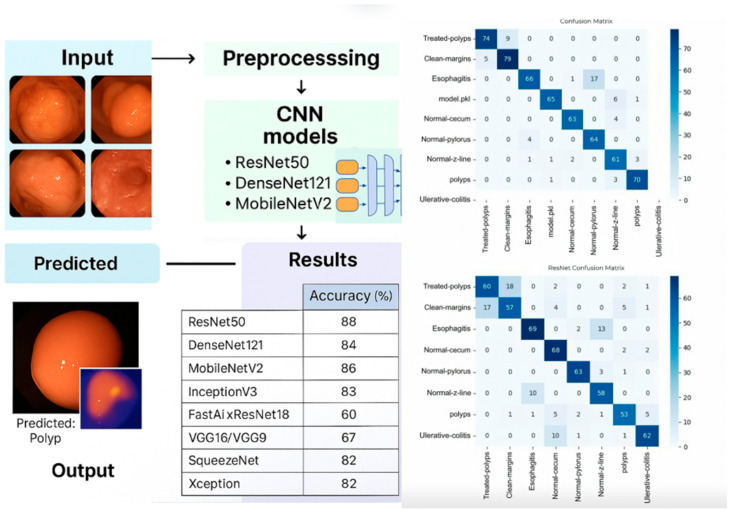
Summary of the proposed workflow for automatic classification of gastrointestinal lesions. The pipeline consists of four core stages: image acquisition, preprocessing, and CNN-based model training.

Figure 1 presents a biologically inspired pipeline for deep learning-based classification of gastrointestinal lesions. The workflow begins with raw endoscopic image acquisition (Kvasir dataset), followed by a preprocessing stage including augmentation and normalization.

Subsequently, CNN models such as ResNet50 [16], DenseNet121 [17], MobileNetV2 [24], and others are trained and fine-tuned to perform multi-class classification of gastrointestinal pathologies. The predicted class labels span eight categories: ‘dyed-lifted-polyps’, ‘dyed-resection-margins’, ‘esophagitis’, ‘normal-cecum’, ‘normal-pylorus’, ‘normal-z-line’, ‘polyps’, and ‘ulcerative-colitis’. These are evaluated using a confusion matrix and a suite of performance metrics to assess classification performance across all classes.

### 2.3. Pre-Trained Models in Endoscopic Imaging

**Transfer learning** is an essential technique in ML, allowing the reuse of knowledge acquired by a model trained on a large dataset (such as ImageNet) [19,20] to solve a new task, typically with a smaller dataset. In medical image classification, Transfer learning is particularly important due to the difficulty of collecting large and balanced datasets. These challenges are further compounded by small sample sizes and class imbalance, which can significantly affect the reliability of classification tasks. The following pre-trained neural network architectures were examined during this study: ResNet50 [16], DenseNet121 [17], MobileNetV2 [24], InceptionV3 [30], Xception [31], VGG16, VGG19 [14], SqueezeNet [32], and FastAI xResNet18 [33].

Residual Network (ResNet) [16] is a deep learning architecture that solves the problem of vanishing gradients through the use of residual connections (or shortcut links). These connections allow data and gradients to flow more easily across layers, enabling effective training of very deep networks. ResNet50 [16] was selected for this study after preliminary tuning and benchmarking trials. It is a specific implementation of the ResNet family, composed of 50 layers, including convolutional, pooling, and fully connected layers. The number 50 refers to the depth of the network. Deeper variants such as ResNet18, ResNet101, and ResNet152 indicate the total layer count and demonstrate stronger feature representation but suffer from increased training time, susceptibility to overfitting, and resource constraints. Lighter models like ResNet18 lacked sufficient feature depth for subtle texture discrimination. This architecture allows the model to learn complex features without losing performance on validation sets.

DenseNet121 [17] connects each layer to all previous layers, ensuring efficient reuse of extracted features and better information propagation. The number 121 refers to the total number of layers, including convolutional, pooling, and fully connected components. This depth supports rich hierarchical representations [12] while maintaining parameter efficiency due to its dense connectivity. Thus, DenseNet121 was chosen as the best architecture, capable of capturing subtle visual differences between gastrointestinal classes with balanced generalization and training performance.

MobileNetV2 [24] is optimized for computational efficiency, employing depthwise separable convolutions and inverted residual blocks. Unlike DenseNet, which encodes its architectural depth directly in its name (e.g., DenseNet121 has 121 layers), MobileNet does not specify layer depth in its naming convention. The term “V2” simply denotes the second major iteration of the MobileNet architecture, rather than indicating network depth [34].

### 2.4. Pre-Trained Architectures and Transfer Learning

All the architectures used in this study were pre-trained on the ImageNet dataset [19,20], which enabled faster and more efficient fine-tuning on the Kvasir dataset. This approach leverages learned representations from large-scale image classification tasks and is particularly valuable when working with smaller or imbalanced medical datasets.

The following CNN architectures were evaluated: InceptionV3—GoogLeNet Inception version 3, VGG16/VGG19—Visual Geometry Group networks with 16 and 19 layers, Xception, Extreme Inception, SqueezeNet—Lightweight CNN with fire modules, FastAI xResNet18—Extended Residual Network with 18 layers. Among these, the most efficient and stable models, based on validation accuracy and reduced overfitting, were ResNet50, DenseNet121, and MobileNetV2.

### 2.5. Inception Architecture

The suffix V3 in InceptionV3 refers to the third major version of the Inception architecture, originally developed by Google. Earlier versions include:

- InceptionV1 (GoogLeNet): Introduced parallel convolutions of varying sizes (1 × 1, 3 × 3, 5 × 5) and auxiliary classifiers to improve gradient flow.
- InceptionV2: Replaced expensive 5 × 5 convolutions with stacked 3 × 3 layers, introduced batch normalization, and improved computational efficiency.
- InceptionV3: Built upon V2 with additional optimisations such as factorised 7 × 7 convolutions, label smoothing, and RMSprop optimisation. It also included deeper modules and more efficient grid size reduction strategies, resulting in improved accuracy and reduced training cost [15].

The selection of deep learning architectures in this study was initially guided by bibliographic research [4,13,17] and confirmed generalization and stability during training in medical image classification tasks. The models ResNet50 with the highest validation accuracy of 90%, followed by DenseNet121 (87.5%) and MobileNetV2 (86.5%), were evaluated through extensive experimentation on the Kvasir dataset.

To ensure comparability, all models were trained and validated using the same dataset split, image preprocessing pipeline, and evaluation metrics, including accuracy, precision, recall, and F1-score. Additionally, the selected models demonstrated excellent compatibility with the development environment used (TensorFlow/Keras), enabling the easy implementation of components such as augmentation and metric monitoring. These architectures benefit from pre-trained versions on ImageNet [19,20], and the use of Transfer learning significantly reduced training time.

### 2.6. Preprocessing Techniques

During the preprocessing stage, images were normalized, converted into values between 0 and 1, and subjected to augmentation operations, including translations, zooming, and horizontal and vertical rotations. These changes assist models in becoming more adaptable and able to recognize things even when they appear in different locations or angles than the training set [27].

The data were pre-processed through normalization, augmentation, and feature extraction to improve model generalization and robustness. For training, the Kvasir dataset was randomly split into three subsets: 70% for training, 15% for validation, and 15% for testing, ensuring that each class was equally represented across all splits. Multiple CNN models, including ResNet50, DenseNet121, MobileNetV2, InceptionV3, VGG16/VGG19, FastAI xResNet18, and Xception, were trained using TensorFlow, Keras, and FastAI frameworks, leveraging state-of-the-art optimization techniques.

The performance of each model was compared, and the best-performing models were tested again to verify their final score. Augmentation allows the model to see multiple variations in the same image, contributing to better learning and reducing the risk of overfitting on the dataset.

In addition to the architectures selected based on a bibliographic review of state-of-the-art CNN models for medical image classification, approximately ten other architectures were evaluated during preliminary experimentation conducted as part of this research. These experimental trials allowed for comparative benchmarking on the Kvasir dataset, enabling the identification of models with the best performance in terms of accuracy, generalization, and computational efficiency. These included NASNetLarge [35,36], EfficientNetB0-B8 [37], ConvNeXt [38], VGG11 [14], InceptionResNetV2 [30], AlexNet [13], ResNet101 [16], DenseNet201 [17], Xception [31] with extensive augmentation, and several custom CNNs [21]. These models were ultimately excluded due to severe overfitting and significant discrepancies between training and validation accuracy. Certain architectures, such as NASNetLarge and EfficientNetB7, performed well on the training set but significantly worse on the validation set, indicating poor generalization to new data (overfitting). Other networks, such as ConvNeXt or DenseNet20, were too complex and required excessive time and memory for training.

## 3. Results: Experimental Results and Performance Analysis

### 3.1. Experimental Environment

All experiments were conducted on a Lenovo IdeaPad Gaming 3 15IAH7 laptop running Windows 11 (64-bit). The system configuration included an Intel Core i5-12500H CPU (12 cores, up to 4.50 GHz), 16 GB DDR4 RAM, and a dedicated NVIDIA GeForce RTX 3050 (4 GB GDDR6). The GPU architecture is based on Ampere, supporting CUDA cores and mixed-precision training Via Tensor Cores. Deep learning libraries such as TensorFlow 2.13, Keras, and DNN were used with GPU acceleration enabled for all CNN models.

### 3.2. Model Training-Validation-Testing

Model Training Duration. Training time ranged from 2 to 4 h per model, depending on architectural depth and internal complexity. Lightweight networks such as MobileNetV2 and SqueezeNet converged within 2 h, while deeper architectures like DenseNet121, ResNet50, and Xception required up to 4 h. These values reflect experiments conducted with optimised batch sizes, early stopping, and adaptive learning rate scheduling.

The development of automatic image classification methods followed experimental steps, where the dataset was split into training (70%), validation (15%), and testing (15%) sets. This distribution provides a good balance between model learning and performance evaluation. In the first stage, the CNN model learns to identify characteristic patterns for each class in the dataset. The training set contains labelled images, and the learning algorithm gradually adjusts the model’s internal weights to reduce classification errors. Training is performed over multiple epochs (between 20 and 50 epochs), and performance is monitored using loss metrics and accuracy. After each training epoch, the models’ performance is evaluated on a validation set. This step checks whether the model generalizes well to new data and prevents overfitting.

As suggested in prior literature [39], adjustments to hyperparameters such as learning rate and batch size can improve model generalization. In the present study, these adjustments were performed iteratively based on the validation score and training dynamics, ensuring optimal convergence during experimentation.

**Figure 2:**
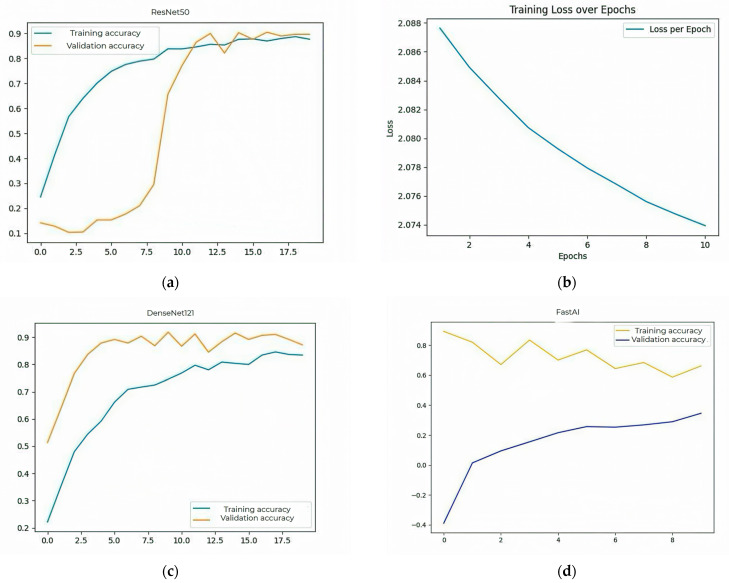

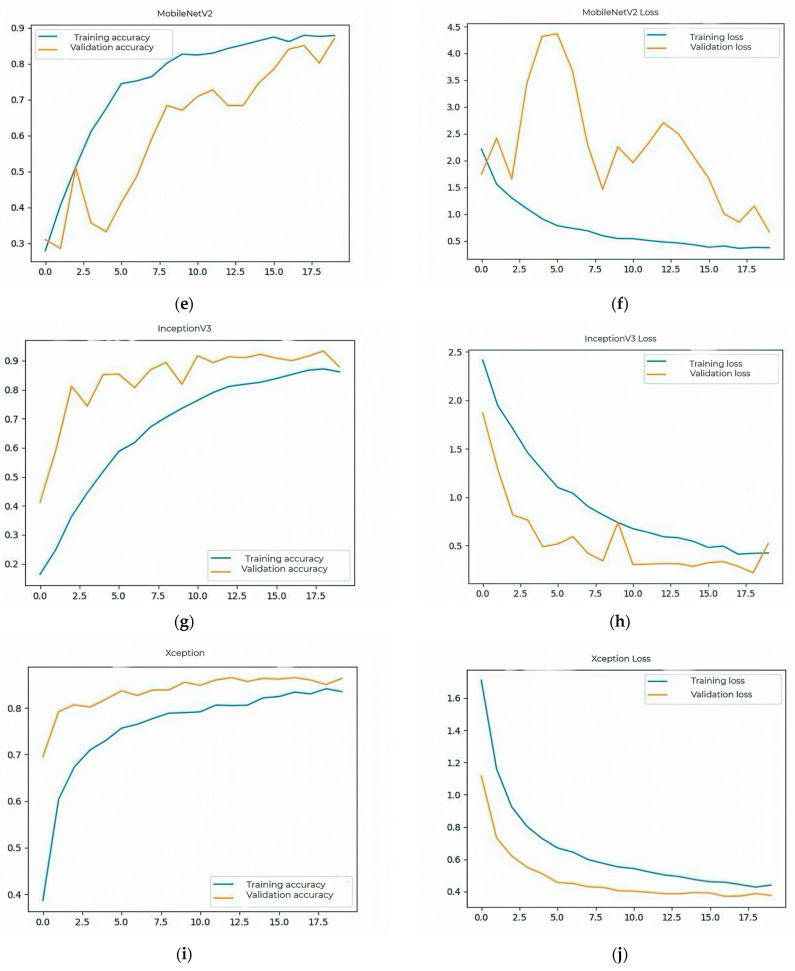

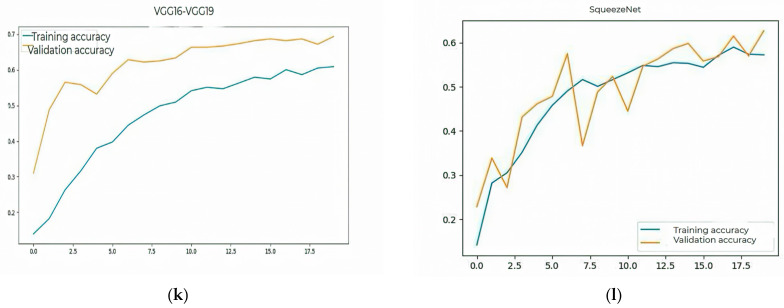
Accuracy network models: (**a**) ResNet50 Accuracy; (**b**) ResNet50 -Training Loss over Epochs; (**c**) DenseNet121 Accuracy; (**d**) FastAI Accuracy; (**e**) MobileNetV2 Accuracy; (**f**) MobileNetV2 Loss; (**g**) InceptionV3 Accuracy; (**h**) InceptionV3 Loss; (**i**) Xception Accuracy; (**j**) Xception Loss; (**k**) VGG16–VGG19 Accuracy; (**l**) SqueezeNet Accuracy. In addition to the final accuracy, other important aspects of each network were also tracked, such as the stability of the curves over time, the difference between the maximum and minimum values during training, and the consistency between epochs. The more stable models, considered ResNet50 and DenseNet121, had linear loss curves without major fluctuations, indicating constant learning. On the other hand, networks with an increased risk for overfitting and instability included VGG16 and SqueezeNet.

Further displays the evaluation metrics, accuracy, and loss plots for each CNN architecture tested. These graphs show the model’s behavior during training and validation. For the ResNet50 model, the performance expressed in accuracy is 88% for the training data and 90.5% on the test data. The graph is plotted in Figure 2a. The plot in Figure 2b shows the categorical cross-entropy loss values computed on the training set across successive epochs for the ResNet50 model. The downward trend reflects effective learning and progressive error minimization during model training.

For the DenseNet121 model, the accuracy obtained during training was 84% and, on the validation set, it reached 87%. This model provided a good balance between performance and computational efficiency. The evolution of the accuracy during training is presented in Figure 2c.

The FastAI model achieved an accuracy of 64% in training and 70% on validation data. Although it has an automated hyperparameter adjustment system, it did not provide competitive results compared to the other models. Its accuracy plot is shown in Figure 2d.

The MobileNetV2 model achieved an accuracy of 86% on training data and 87% on validation data. Due to its small size and low resource consumption, it is suitable for deployments on devices with limited computing power. Figure 2e illustrates the accuracy, and Figure 2f shows the corresponding losses.

InceptionV3 provided an accuracy of 83% on the training set and 85% on the validation set. This model was distinguished by its ability to learn features at multiple scales, but the training time was longer. Figure 2g,h shows the accuracy and loss plots.

The Xception model achieved an accuracy of 82% on training and 85% on validation. Due to its architecture based on convolution separation, it provided a good balance between speed and performance. Figure 2i,j shows the evolution of accuracy and loss.

The accuracy obtained by the VGG16 and VGG19 models was 60% on training and 68% on validation. These architectures have a simple structure, but the large number of parameters led to a pronounced overfitting tendency. Their plot is represented in Figure 2k. SqueezeNet had an accuracy of 57% on training and 63% on validation. Although extremely compact and fast, the model failed to capture essential features of medical images sufficiently well. Figure 2l shows the accuracy plot.

### 3.3. Evaluation Methodology: Accuracy, Confusion Matrix, and Inference Time

Model performance evaluation is conducted using multiple classification metrics to better understand the behaviour of the CNNs, such as ResNet50, DenseNet121, and MobileNetV2 on unseen data from the test set. After training and validation, the final step is model testing. The model is evaluated on a test set composed of unseen images to simulate its performance in real scenarios. The following evaluation metrics were calculated during this stage: Confusion Matrix, Accuracy, Precision, Sensitivity (Recall), and F1-score for each class. These metrics were chosen to identify different aspects of model performance for multi-class medical image classification, where hard example reweighting and clinical risk make single-metric evaluation insufficient.

Confusion Matrix visualises misclassifications across all classes. It reveals patterns such as false positives or confusion between visually similar polyp types, guiding further refinement or reannotation. Accuracy provides a general overview of correct classifications but can be misleading when classes are imbalanced. For example, if one polyp type is overrepresented, a high accuracy may mask poor performance on rare classes. Precision reflects the proportion of true positives among predicted positives for each class. In clinical settings, high precision is crucial to minimise false diagnoses. Sensitivity (Recall) indicates the proportion of true positives detected among all actual instances of a class. This is especially important in medicine, where failing to detect a pathology (false negative) can be more dangerous than over-detection. F1-score balances precision and recall. It is particularly valuable when the dataset is unbalanced or when both false positives and false negatives carry clinical risk.

While all metrics contribute to a comprehensive evaluation, Sensitivity and F1-score are often considered more critical in medical imaging classification. This is because missing a lesion or polyp (false negative) may have direct implications for diagnosis and treatment outcomes.

### 3.4. Model Comparison Inference

Once trained, most models demonstrated rapid inference performance. Specifically, MobileNetV2, DenseNet121, and ResNet50 consistently processed individual images in less than 1 s, even on consumer-grade hardware without TPU or multi-GPU setups. Heavier models such as EfficientNetB7, NASNetLarge, and InceptionResNetV2 exceeded 1 s per image, making them unsuitable for real-time deployment under constrained resources.

For each model, the time required to process a single image was measured. On average, inference is completed in less than one second, demonstrating the accessibility of these models even on limited hardware. It can be observed that the model has a high classification rate for some classes but frequent misclassifications between others. The inference time averaged under 0.5 s per image, even on consumer-grade hardware.

The ResNet50 model obtained the highest accuracy on the validation set, followed by MobileNetV2 and DenseNet121, according to the findings in Table 1. The models’ performance was compared and evaluated using a confusion matrix and accuracy analyses. Bolded values indicate the highest performance per column. Although the training time was significant (between 2 and 4 h per model), the results were stable, and the differences between training and validation accuracy were small in the case of well-optimised models. This indicates good generalisation capability.

### 3.5. Biomimetic Behavior Analysis

In Table 2 we analysed model behavior through training and validation curves to assess biomimetic principles: Feature Reuse (DenseNet121): The minimal gap between training and validation accuracy suggests efficient abstraction and reduced overfitting;Residual Connections (ResNet50): Smooth convergence and stable loss trends reflect layered abstraction and gradient stability; and Attention Mechanisms (CapsuleNet): Higher stability is indicated by better performance on classes with shape variation, such as polyps.

We compared our models with commercially available diagnostic systems, including Fujifilm CAD EYE [14] and Olympus ENDO-AID [40], which are integrated into endoscopy platforms and deployed in clinical environments. These tools serve as benchmarks for real-time polyp detection and classification. Table 3 presents a comparative overview of our models and the referenced commercial systems in terms of accuracy, inference time, parameter count, explainability, and cost efficiency. It is important to note that this numerical comparison is intended for orientation purposes only, as the models were evaluated on different datasets and experiments.

### 3.6. Confusion Matrix Interpretation

To evaluate the performance of each model, the confusion matrix was used. This matrix provides insights into the best and worst classified classes [41], illustrating the classification performance of two deep learning models on gastrointestinal image data. Each matrix displays the number of correct and incorrect predictions per class, with darker shades indicating higher counts. Diagonal cells represent correct classifications, while off-diagonal cells indicate misclassifications. These visualizations support the evaluation of model robustness and class-specific accuracy.

**Figure 3:**
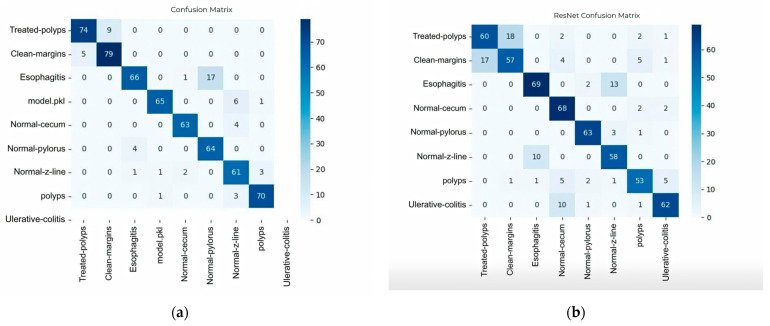
Confusion matrices: (**a**) Confusion matrix for the first classification model; (**b**) Confusion matrix for the ResNet model.

For the first classification model, the Confusion matrix can be seen in Figure 3a, evaluated on a distinct dataset with nine gastrointestinal classes. The matrix reveals high accuracy for bleed-polyps, clean-margins, and model.pkl, with minor confusion between esophagitis and small-cecum. The presence of the model.pkl label indicates a placeholder or mislabeled class, which may require clarification or exclusion in future iterations.

For the ResNet model in Figure 3b, the Confusion matrix shows classification results across eight gastrointestinal categories. The matrix highlights strong performance on classes such as detected-polyps, esophagitis, and ulcerative-colitis, with most predictions concentrated along the diagonal. Misclassifications are minimal and primarily occur between visually similar categories, such as detected-polyps and non-margins. The ratio of correct predictions to the total number of tested examples provides an initial overview of model efficiency, making it easy to interpret. However, when dealing with imbalanced classes, accuracy can be misleading; therefore, it must be complemented by other metrics [42].

The right-side values are most likely the color bars; they define the range of values used in the heatmap shading. Each cell in the confusion matrix represents the count of predictions (e.g., the number of samples from class A that were incorrectly predicted as class B). The heatmap uses colors to visualize those counts. The values on the right −20, 0, …, 70 correspond to color intensity, not the actual data. The negative value (−20) likely suggests either an artifact in normalization, scaling, or it’s a misconfigured threshold, since confusion matrices usually don’t include negative values.

As can be observed, the model that performed the best in this analysis, ResNet50, demonstrates confusion between visually similar classes while reaching high classification rates for some of them. Overlapping textures or color profiles, especially between polyps and inflamed mucosa, may be the cause of these misclassifications. On the other hand, visually recognized and well-represented classifications like normal or ulcerative-colitis have consistently shown good precision and recall ratings.

## 4. Discussion

Our results imply that architectural improvement may reduce the requirement for additional data. The biomimetic design method was validated by DenseNet121 and CapsuleNet, which demonstrated strong results on both sparse and complex classes. Hard instances were successfully handled by the dynamic revision technique, which increased model stability without changing class frequencies. The dataset split was explained as follows: 70% for training, 15% for validation, and 15% for testing. The model was chosen based on the smallest validation loss, and Table 1 results relate to the test set.

**Figure A1:**
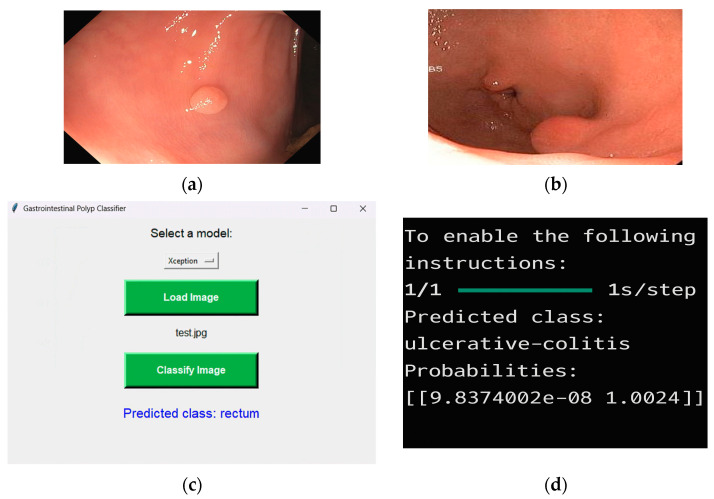
Graphical interface evaluation and real-image testing: (**a**,**b**) Endoscopic images of a real gastrointestinal polyp used for testing and validating model prediction in real-world conditions; (**c**) Custom-built GUI for gastrointestinal polyp classification using the Xception model. The interface includes model selection, image input, and output prediction functionality, in this instance, displaying predicted class: rectum; (**d**) Image-based inference.

The GUI implementation and low inference latency (<0.5 s), mentioned in Figure A1, support real-time deployment in clinical settings, similar to commercial systems such as Olympus ENDO-AID and Fujifilm CAD EYE. Our models, in contrast to these proprietary systems, have fewer resource needs, better explainability thanks to Grad-CAM integration, and comparable accuracy. 

### 4.1. Improvements

To improve CNN model performance on gastrointestinal polyp classification from endoscopic images, the study implemented several targeted:

- Customised Data Augmentation: While augmentation is a standard technique, this study applies an augmentation pipeline, including rotation, scaling, flipping, and contrast adjustment, optimised for gastrointestinal polyp morphology. This approach reduces overfitting.
- Dynamic Class: A novel weighting scheme was implemented based on real-time class distribution during training, rather than static frequency-based weights. This improves learning stability across imbalanced classes.
- Explainability: Grad-CAM was integrated as a feedback mechanism during model refinement. This dual use helped identify misclassified regions and guided architectural adjustments.

While VGG16, SqueezeNet, and NASNetLarge showed promising learning rates early in training, extensive augmentation and architectural regularisation were critical to overcoming their overfitting behavior.

### 4.2. Error Analysis

Errors during CNN training were classified into two major types:

- Confusions between visually similar classes: The most frequent misclassifications involved visually similar classes, such as polyps and inflamed mucosa, which shared overlapping color gradients, mucosal textures, and ill-defined boundaries.
- Hard example reweighting Sensitivity: These include inter-class confusion (e.g., misidentifying hyperplastic polyps as adenomatous ones) and incorrect localisation or attention to irrelevant image regions.

Precision and recall were lower for classes with fewer representative samples, such as uncommon polyp subtypes. This results from bias caused during training. These classification challenges were compounded by limitations in dataset diversity, inconsistent lighting, and the absence of pixel-level annotations. Solutions may include using advanced sampling strategies, synthetic data generation, or incorporating spatial attention mechanisms to focus learning on relevant image regions.

### 4.3. Limitations

Despite rigorous experimentation, the study encountered several constraints:

- Dataset Size: The Kvasir dataset contains a relatively limited number of images for certain polyp subtypes, which can impair generalisation and lead to classifier bias.
- Overfitting in Complex Models: Architectures like EfficientNetB8, InceptionResNetV2, and NASNetLarge demonstrated high variance between training and validation metrics, indicating overfitting. These models were excluded from the final evaluation.
- Domain Limitation: The trained models performed well on Kvasir data, but may experience performance degradation when applied to endoscopic images from different institutions, due to lighting conditions, device variability, and annotation inconsistency.
- Hardware Constraints: Training and tuning were conducted on an RTX 3050 GPU (4 GB), which limited the ability to perform extensive hyperparameter tuning or ensemble testing across large architectures.

### 4.4. Proposals for Future Integration with Segmentation and Multi-Label Classification

This work focused exclusively on image classification, but the following directions for future development could be considered:

- Integration of a segmentation component: where the model not only classifies but also highlights the affected area in the image. This could be achieved by U-Net or Mask R-CNN-type models, which would add a significant plus to the system.
- Multi-label classification: multiple features or lesion types may appear in some images, so future models should be trained to recognize multiple classes simultaneously in a single image.
- Creation of a graphical user interface: allowing the clinician to load an image and obtain an instant prediction, with the option to visualize the probability of each class.
- Model Explainability: to increase the confidence of physicians, techniques such as Grad-CAM could be integrated to visually show which region in the image was the basis for the network decision.
- Use of Vision Transformer (ViT) models: future work could investigate the replacement of CNNs with ViTs, attention-based models that provide competitive results in medical imaging, especially in fine lesion detection [43].
- Deployment of generative models (GANs): GANs can be used to generate synthetic medical images, increasing training sets and improving model generalization, especially in sparse or imbalanced classes [44].

Recommended solutions to address classification challenges included the use of advanced data augmentation strategies [2,26], such as rotation, flipping, contrast adjustment, and scaling, to artificially increase dataset diversity and improve model generalisation. Additionally, explainability techniques were applied, most notably *Gradient-weighted Class Activation Mapping (Grad-CAM)*, which generates visual heatmaps to highlight the regions within an image that influenced the model’s prediction. These methods help identify potential sources of error and improve transparency, making CNN-based decision systems more interpretable for clinicians.

## 5. Conclusions

In this study, a biomimetic Deep Learning-image classification model based on CNNs was proposed to automatically identify GI lesions from endoscopic images. Several deep learning models for automatic classification of gastrointestinal polyps in endoscopic images were compared. It was performed to identify the architecture that provides the best results in accuracy and generalization ability. Actual state-of-the-art CNN models such as ResNet50 [16], DenseNet121 [17], MobileNetV2 [24] were used for training. Moreover, other architectures were also tested; some returned worse results due to overfitting.

Results show that the ResNet50 model obtained the best accuracy on the validation set, followed by MobileNetV2 and DenseNet121. The models were evaluated by accuracy and confusion matrix, and their performances were compared in the previous chapter.

To assist users such as physicians, in Figure A1d, an interface was also designed that allows the selection of one of the trained models mentioned above. The interface allows the user to select an image that they wish to assign to a class and visualize which class the image belongs to, based on the previously selected model.

Even though the training time was significant (between 2 and 4 h for each model), some stable results were obtained, and the differences between the accuracy on the training and validation sets were small for the well-optimized models, indicating good generalization.

In practice, the models performed well even on consumer-grade hardware, being tested on a Lenovo IdeaPad Gaming 3 laptop (RTX 3050, i5-12500H). The average inference time for an image was under one second, allowing these systems to be integrated into a real-time workflow without significant differences.

It was observed that most classifications were correct, especially for classes well represented in the training data. Classes with large variations or few examples were harder to classify, indicating the need for additional data.

In conclusion, this research presents a biomimetic AI-based pipeline for medical image classification, demonstrating potential for aiding colorectal cancer screening and improving diagnostics in clinical environments.

## Figures and Tables

**Table 1:** Performance comparison of CNN models on the Kvasir test set: Accuracy, Precision, Recall, and F1-score.

Model	Train	Valid	Prec	Rec	F1	Predicted Class
ResNet50 DenseNet121 MobileNetV2	**88%**	**90%**	**92%**	**89%**	**90.5%**	dyed-lifted-polyps
	84%	87%	89%	86%	87.5%	dyed-resection-margins
	86%	87%	88%	85%	86.5%	esophagitis
InceptionV3 FastAI xResNet18	83%	85%	86%	83%	84.5%	normal-cecum
	64%	70%	74%	68%	71.0%	normal-pylorus
VGG16/VGG19 SqueezeNet Xception	60%	68%	70%	63%	66.0%	normal-z-line
	57%	63%	68%	59%	63.0%	polyps
	82%	85%	85%	84%	84.5%	ulcerative-colitis

**Table 2:** Comparative table of architectures.

Model	Accuracy	Training Time	Params (M)	Inference Speed (ms)	Misclass. Rate
DenseNet121 ResNet50 MobileNet	92.3%	3 h 20 m	8.0	45	7.1%
	91.8%	2 h 50 m	25.6	52	7.6%
	90.2%	2 h 15 m	4.2	35	8.0%
CapsuleNet	89.5%	4 h 10 m	11.2	60	8.3%

**Table 3:** Comparative performance of proposed models versus commercial diagnostic tools. Values are approximate and intended for orientation only due to dataset differences.

Model	Accuracy	Inference Time	Parameters	Explainability	Cost Efficiency
ResNet50 DenseNet121 CapsuleNet	90%	0.45 s	25 M	Grad-CAM	High
	92%	0.48 s	8 M	Grad-CAM	High
	88%	0.60 s	12 M	Built-in	Moderate
ENDO-AID and CAD EYE	85–87%	Real-time	Proprietary	Limited	High

## Data Availability

The original contributions presented in this study are included in the article. Further inquiries can be directed to the corresponding authors. The dataset consists of Simula Research Laboratory, Kvasir Dataset, https://datasets.simula.no/kvasir/ (accessed on 8 February 2025). These data originate from real colonoscopy procedures, providing authentic and clinically relevant visual and feature-based information for research and analysis.

## Appendix A

### Testing the ResNet50 Model Using a Graphical User Interface (GUI)

Trained models could be a tool for gastroenterologists, especially in endoscopic diagnostic procedures. A rapid classification of polyps could make early detection of precancerous lesions easier, thereby reducing the risk of colorectal cancer development [1,2]. Figure A1 shows the very simplified graphical user interface that has been built to help users, such as physicians, test the model. The interface allows the user to select one of the trained models for predictions. For predictions, the *Load Image* button needs to be clicked to select the image to be classified. Pressing the *Start Classification* button returns the class of the loaded image, using the model chosen by the user. The chosen model (ResNet50) was successful in predicting the image’s class in Figure A1a,b. According to the model, the image represents a polyp that belongs to the ulcer class, as predicted by the model.

The result of a classification can be seen in Figure A1. The selection of the trained models is an advantage for the clinician, as they can make a comparison to return an accurate prediction. The model loads an image, processes an image, and generates the model prediction in a very short time (1 s/step), displaying the estimated class as shown in Figure A1d, thus highlighting the practical applicability.
